# A Simple Electrochemical Route to Access Amorphous Co-Ni Hydroxide for Non-enzymatic Glucose Sensing

**DOI:** 10.1186/s11671-019-2966-2

**Published:** 2019-04-17

**Authors:** Hongbo Li, Ling Zhang, Yiwu Mao, Chengwei Wen, Peng Zhao

**Affiliations:** Institute of Nuclear Physics and Chemistry (INPC), China Academy of Engineering Physics (CAEP), Mianyang, 621999 People’s Republic of China

**Keywords:** Amorphous, Co-Ni hydroxide, Glucose sensor, Non-enzymatic

## Abstract

Among the numerous transition metal hydroxide materials, cobalt- and nickel-based hydroxides have been extensively studied for their excellent electrochemical performances such as non-enzymatic electrochemical sensors. Binary cobalt-nickel hydroxide has received extensive attention for its exceptionally splendid electrochemical behaviors as a promising glucose sensor material. In this work, we report the synthesis of three-dimensional amorphous Co-Ni hydroxide nanostructures with homogeneous distribution of elements via a simple and chemically clean electrochemical deposition method. The amorphous Co-Ni hydroxide, as a non-enzymatic glucose sensor material, exhibits a superior biosensing performance toward glucose detection for its superior electron transfer capability, high specific surface area, and abundant intrinsic redox couples of Ni^2+^/Ni^3+^ and Co^2+^/Co^3+^/Co^4+^ ions. The as-synthesized amorphous Co-Ni hydroxide holds great potential in glucose monitoring and detection as non-enzymatic glucose sensors with high sensitivity 1911.5 μA mM^−1^ cm^−2^ at low concentration, wide linear range of 0.00025–1 mM and 1–5 mM, low detection limit of 0.127 μM, super long-term stability, and excellent selectivity in 0.5 M NaOH solution.

## Introduction

Carbohydrate, as one of the most important energy sources, can be used to evaluate the healthy condition of the human body by monitoring sugar level in blood. Diabetes mellitus or diabetes, a clinical chronic life-threatening disease caused by an elevated level of glucose in the blood, has become a global universal epidemic. Diabetes can be classified into two types based on their controlled mechanism: type 1 caused by inadequate insulin production in the body and type 2 governed by the body’s inability to use its produced insulin [1]. Because the essential for the proper diagnosis and treatment of diabetes is to monitor and control the physiological glucose level precisely, continuous monitoring of physiological glucose levels with high accuracy and fast response has become a potential trend. The urgent requirements of glucose detection devices attract extensive attention to focus on the design and development of novel glucose sensors with high accuracy and sensitivity, low cost, fast response, excellent selectivity, and reliability. According to the transducing mechanism, glucose detection devices can be categorized into a series of potential strategies such as a resonator, field-effect transistor, optical detector, and electrochemical sensor [2–6]. Among of them, the electrochemical sensors have been recognized as the most promising glucose sensors with the several attractive features of being stable, inexpensive, implantable, portable, and miniaturize for their fast, accurate, and reliable route to determine the glucose concentration [6, 7]. Based on the sensing mechanism, electrochemical glucose sensors can be classified into two species: enzyme-based biosensors and non-enzymatic sensors [2]. Because of poor stability, high cost, and easy denaturation of enzyme-based biosensors, extensive efforts have been devoted on the development of novel non-enzymatic electrochemical glucose sensors for high sensitivity, long-term stability, low cost, and facile fabrication [8].

Thus far, the development in non-enzymatic electrochemical glucose sensors has significantly progressed using some original nanomaterials, such as pure metal [9–11], metal oxide [12, 13], and carbon-based composite [14]. Among numerous non-enzymatic electrochemical sensors, non-noble transition metal (such as Ni [15] and Cu [16]) and metal oxide (such as NiO_X_/Ni(OH)_2_/NiOOH [17–19], CoO_x_ Co(OH)_2_/ CoOOH [20–22], CuO_x_ [23], and ZnO_X_ [13]) have been demonstrated as promising active materials for non-enzymatic glucose sensors with low cost and abundance in earth compared with that of noble metal-based materials. The sensing performance of non-enzyme glucose sensors is significantly controlled by the morphology, microstructure, and composition of nanomaterials. The single metal or metal oxide-based materials have exhibited limited potential for their unitary composition. According to previous studies, it can be found that the multi-metal alloy or multi-metallic compounds greatly promote the integrated electrochemical performance. Recently, more and more attention has been focused on designing and fabricating binary metal or bimetallic oxide composite such as Co-Ni [24], Ni-Fe [25], and Ni-Cu [26] for their diversity in preparing bimetallic compositions and flexibility in forming complex three-dimensional (3D) structures resulting in superior electrochemical activity for glucose sensing. The bimetallic compound electrochemical sensors of Ni-Co-based materials receive increasing attention for their advanced electrocatalytic properties and chemical stability [24, 27–29].

Most of the active materials that have been widely applied to non-enzymatic glucose sensors are based on crystalline phase. Amorphous phase materials are previously assessed as unsuitable electrochemical glucose sensors for their poor electrochemical performance [30]. However, the amorphous phase has been recently demonstrated to possess impressive electrocatalytic behavior, which may be exploitable in certain device applications [31, 32]. Considering the excellent electrochemical glucose sensing performance of crystalline bimetallic hydroxide electrodes, several benefits should be realizable by the development of amorphous bimetallic hydroxide sensors. Here, we focus our attention on the advantages of the amorphous phase in non-enzymatic electrochemical glucose sensors based on amorphous Co-Ni hydroxide, prepared by a simple, facile, and chemically clean electrochemical cathode deposition technique. This study is aimed to explore the biosensing performance of the prepared amorphous Co-Ni hydroxide toward glucose oxidation in alkaline solution.

## Methods

### Synthesis of Amorphous Co-Ni Hydroxide Nanostructures/Graphite Electrode

The amorphous Co-Ni hydroxide nanostructures were fabricated in one step by an idiographic electrochemical cathode deposition method. In detail, the prepared amorphous products were deposited onto a cathodic graphite electrode under the application of a working voltage of 90 V for 12 h from a quartz deposition bath. The reaction bath contains three parts: two parallel graphite sheets as the cathode and anode, both with a working area of approximately 15 mm × 7 mm in size; high-purity de-ionized water as the electrolyte; and a transition Ni-Co alloy (Ni/Co molar ratio of 1:1) target of 20 mm × 20 mm × 20 mm in size at the center of the bath floor as metal ion source.

### Characterization of Amorphous Co-Ni Hydroxide Nanostructures

Scanning electron microscope (SEM) and transmission electron microscope (TEM) were carried out on a JSM-7600F SEM at an accelerating voltage of 15 kV and FEI Tecnai G2 F30 TEM at an accelerating voltage of 300 kV, respectively, to identify the morphology, crystallinity, and microstructure of the as-synthesized amorphous glucose sensors [33, 34]. The energy-filter TEM mapping was employed to analyze the element distribution of the amorphous products. In addition, the surface chemical states of the bonded elements of the products were characterized by using an ESCA Lab250 X-ray photoelectron spectroscopy (XPS) and an argon-ion laser micro-Raman spectroscopy (Renishaw inVia, 785 nm).

### Electrochemical Measurements

All electrochemical measurements were conducted using a CHI-760E electrochemical workstation in a typical three-electrode setup with 0.5 M NaOH solution as electrolyte, amorphous Co-Ni hydroxide/graphite as the working electrode, saturated calomel electrode (SCE) as the reference electrode, and platinum wire as the counter electrode.

## Results and Discussions

### Characterizations of the Amorphous Co-Ni Hydroxide Nanostructures

**Fig. 1:**
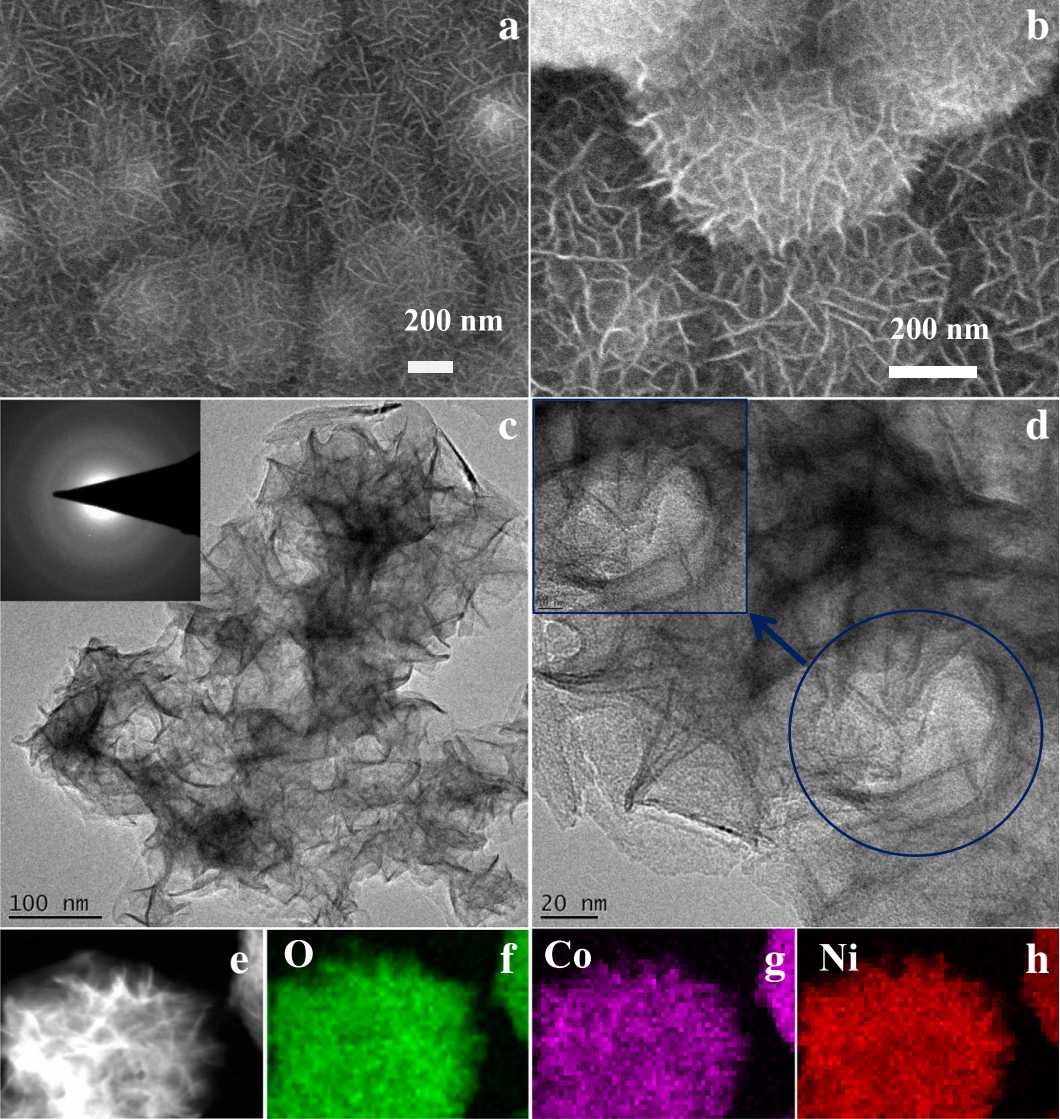
Morphology and structure of the products. **a**, **b** SEM images of the amorphous Co-Ni hydroxide deposited on a graphite sheet. **c**, **d** TEM images of the amorphous Co-Ni hydroxide sample (the insets show the corresponding SAED pattern and HRTEM image). **e** STEM image. **f**–**h** Element mappings of O, Co, and Ni

A series of physical characterizations were carried out to confirm the formation of amorphous Co-Ni hydroxide nanostructures on the graphite substrate. The surface morphology of the modified electrode was characterized by SEM and TEM images as shown in Fig. 1. The amorphous Co-Ni hydroxide nanostructures were successfully fabricated on the surface of the graphite substrate. SEM images (Fig. 1a, b) of as-prepared products show that the nanostructures display a dominant diameter of ~ 400 nm with wrinkled surfaces, revealing a three-dimensional surface. In order to characterize the detailed morphology and structure of the as-synthesized samples, typical low-magnification and high-resolution TEM (HRTEM) images of nanostructures prepared are depicted in Fig. 1c and d, respectively. No evident crystal lattice fringe can be observed in the high-resolution TEM image, so no crystalline morphology was generated in this process. Furthermore, the corresponding selected area electron diffraction (SAED) pattern was investigated as shown in the inset of Fig. 1c and a broad and diffused halo ring can be observed suggesting an amorphous nature [35]. The composition distribution of the as-synthesized nanostructures was investigated by using the element mapping technique as shown in Fig. 1f–h. The element mapping analysis results suggest a highly homogeneous distribution of O (Fig. 1f), Co (Fig. 1g), and Ni (Fig. 1h) in the products, implying the well-homogeneous structure of amorphous Co-Ni hydroxide nanostructures [36].

**Fig. 2:**
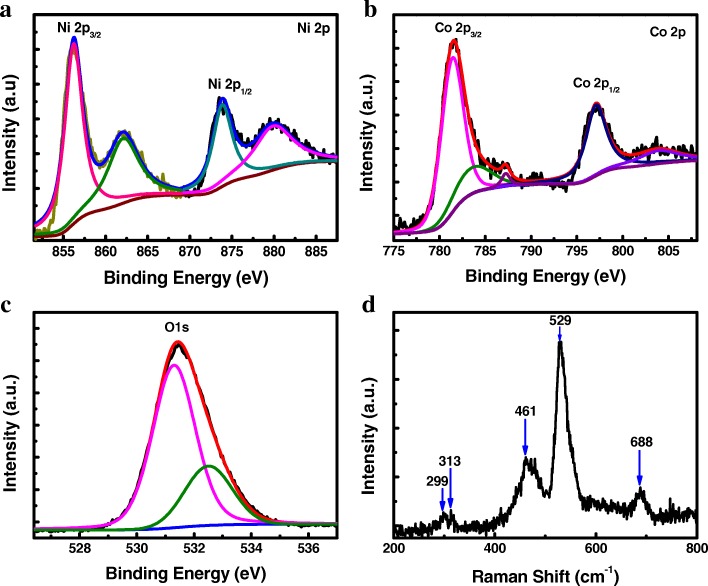
Chemical states of bonded element characterizations of the products. **a**–**c** XPS spectra of Ni 2p, Co 2p, and O 1s. **d** The Raman spectrum of the amorphous Co-Ni hydroxide

XPS, a reliable technique for phase composition detection, was used to investigate the atoms’ chemical states of the modified electrode surface by measuring the binding energy. The typical XPS spectra of Ni 2p, Co 2p, and O 1s with fitting curves by using a Gaussian fitting method are shown in Fig. 2a, b, and c, respectively. The high-resolution Ni 2p spectrum displays that two spin-orbit doublets corresponding shake-up satellite peaks can be seen over the range of 851–888 eV. The fitting results show that two strong peaks with binding energies appeared at 856.3 and 873.9 eV characteristic peaks of the Ni 2p_3/2_ and Ni 2p_1/2_ spin-orbit doublets, respectively, and two corresponding shake-up satellite peaks at 862.2 and 880.1 eV for Ni 2p_3/2_ and Ni 2p_1/2_. These results suggest that the Ni species in the prepared samples are in the +2 oxidation state [18]. Furthermore, the shape of spectrum and the spin-energy separation of 17.6 eV are the characteristics of the Ni(OH)_2_ phase, which is in good agreement with previous reports [18, 37]. Meanwhile, the fitted high-resolution Co 2p spectrum shows the spin-orbit splitting of Co 2p_3/2_ at 781.5 eV corresponding shake-up satellite peaks at 787.2 eV and Co 2p_1/2_ at 797.1 eV corresponding shake-up satellite peaks at 804.2 eV, indicating the +2 oxidation state of Co in the prepared product [38, 39]. In addition, the O 1s spectrum with a strong peak at the binding energy of 531 eV can be associated with bound hydroxyl ion (OH^-^), confirming the formation of M–OH (M = Co, Ni) [18, 37–39]. Moreover, the atomic ratio of Co to Ni is close to 1:1 from the XPS analysis.

Raman spectrum was employed to collect more information about the surface functional groups of the product as shown in Fig. 2d. Two strong broad peaks located at 461 and 529 cm^−1^ and three weak peaks at 299, 313, and 688 cm^−1^ can be observed in the Raman spectrum of Co–Ni(OH)_2_. In particular, the peaks at 299, 461, and 688 cm^−1^ can be indicative of the Co(OH)_2_ phase [39] and the peaks at 313, 461, and 688 cm^−1^ are characteristic of Ni(OH)_2_ phase [40]. The strong bands at 461 and 529 cm^−1^, shifted and broadened, may come from the combination of symmetric Ni–OH/Co–OH stretching mode and the symmetric O–Ni–O/O–Co–O stretching mode, respectively. The band at 313 cm^−1^ can be attributed to the E_g(T)_ mode for Ni(OH)_2_ phase. The peaks of 299, 689, and 191 cm^−1^ may have resulted from the E_g_ and A_1g_ symmetric stretching mode for Co(OH)_2_ phase, respectively. In summary, the characterization results of morphology and structure, obtained from SEM, TEM, SAED, XPS, and Raman measurements, reveal that the amorphous Co–Ni(OH)_2_ nanostructures with irregular and wrinkled surface features were synthesized successfully.

### Electrochemical Performance of Amorphous Co-Ni Hydroxide Electrode

**Fig. 3:**
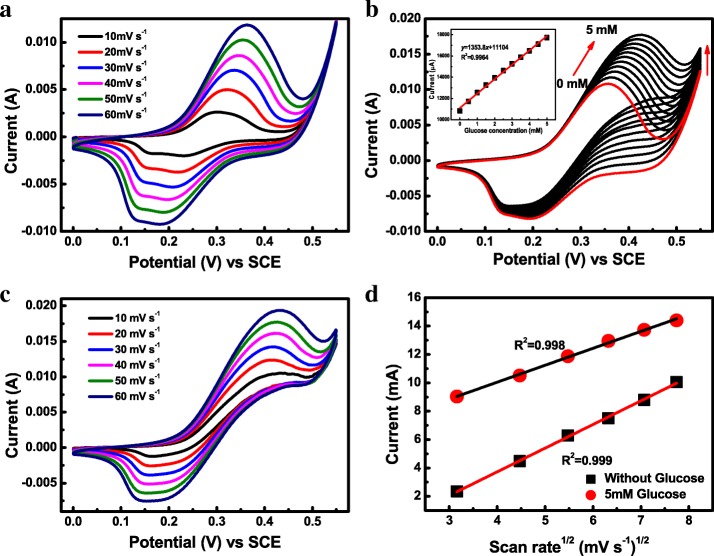
Electrochemical behaviors of amorphous Co-Ni hydroxide toward glucose oxidation in 0.5 M NaOH. **a** CV curves at various scan rates in the absence of glucose. **b** CV curves of the Co-Ni hydroxide electrode with different concentrations of glucose at a scan rate of 50 mV s^−1^. **c** The CV curves of glucose oxidation at different scan rates from 10 to 60 mV in the presence of 5 mM glucose. **d** The fitting plots of *I*_pa_-*ν*^1/2^ in the absence and presence of glucose

In order to obtain an activated and stabilized electrochemical performance, the amorphous Co-Ni hydroxide electrode was firstly scanned at a scan rate of 50 mV s^−1^ in 0.5 M NaOH electrolyte until the cyclic voltammetry (CV) curves overlapped absolutely. Afterward, the CV technique was used to investigate the electrochemical behaviors of amorphous Co-Ni hydroxide electrode in 0.5 M NaOH electrolyte without the glucose addition at various scan rates in the potential window of 0.0 and 0.55 V vs SCE. As shown in Fig. 3a, CV curves of amorphous Co-Ni hydroxide exhibit typical pseudocapacitive behavior for the pair of well-defined quasi-reversible redox couple peaks, specifying the reversible conversion of Ni^2+^/Ni^3+^ and Co^2+^/Co^3+^/Co^4+^ [41]. For example, amorphous Co-Ni hydroxide electrode exhibits a broad strong anodic peak centered at about 0.36 V vs SCE at a scan rate of 50 mV s^−1^, which could be attributed to the different complex oxidation states of Ni and Co. In detail, the Ni^2+^ and Co^2+^ ions were transformed into Ni^3+^ and Co^3+^ ions, respectively, and then, the Co^3+^ ion was further oxidized into Co^4+^ ion at higher potentials. Under the reverse scan, two board cathodic peaks centered at 0.19 and 0.14 V vs SCE were observed at a scan rate of 50 mV s^−1^, corresponding to the reduction of Ni^3+^/Ni^2+^, Co^4+^/Co^3+^, and Co^3+^/Co^2+^, respectively.

**Fig. 4:**
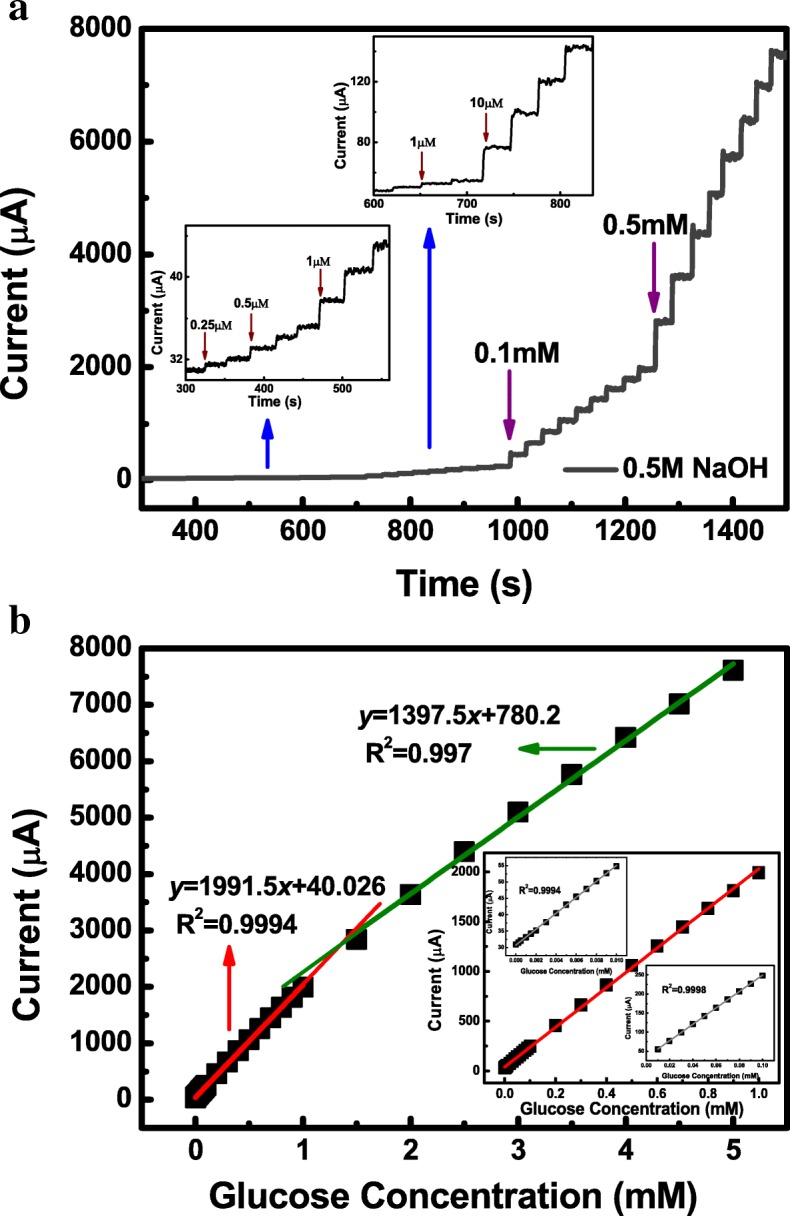
Amperometric detection of glucose. Amperometric current-time (i-t) curve (**a**) and corresponding calibration curve (**b**) of the glucose oxidation on the Co-Ni hydroxide electrode acquired in 0.5 M NaOH

With increasing scan rate, the value of redox peaks current increases gradually, whereas the potentials of the anodic peak (*E*_pa_) and cathodic peak (*E*_pc_) undergo positive and negative shifts, respectively. These phenomena could be attributed to the internal resistance of the amorphous Co-Ni hydroxide electrode. As shown in Fig. 4d, the anode peak current (*I*_pa_) of the amorphous Co-Ni hydroxide electrode in 0.5 M NaOH solution as a function of the square root of scan rate (*ν*^1/2^) was depicted in the absence of glucose. The fitting result reveals that *I*_pa_ performed a linear relationship with *ν*^1/2^ with a high correlation coefficient of 0.999 under the alkaline conditions, suggesting that electrochemical kinetic mechanism for Co-Ni hydroxide electrode is a diffusion controlled process.

### Voltammetric Behavior of Co-Ni Hydroxide Toward Glucose Oxidation

The electrochemical behavior of amorphous Co-Ni hydroxide toward glucose oxidation under the alkaline conditions was further explored by the CV technique. Figure 3b depicts the typical CV curves of the as-prepared Co-Ni hydroxide electrode as a function of glucose concentration ranging from 0–5 mM in 0.5 M NaOH solution at a scan rate of 50 mV s^−1^. As can be seen, upon the addition of glucose, the anodic peak potential shifted positively and the anodic peak currents *I*_pa_ were enhanced gradually, which lays out a prospect for the subsequent quantitative analysis. All the CV curves in Fig. 3b exhibit broad strong anodic peaks which mainly attribute to the oxidation of Ni^2+^/Ni^3+^ and Co^2+^/Co^3+^/Co^4+^ at first. Then, the analyze glucose (C_6_H_12_O_6_) is oxidized into gluconolactone (C_6_H_10_O_6_) through Ni^3+^ and Co^4+^ in alkali electrolyte. Simultaneously, NiO(OH) and CoO_2_ are reduced into Ni(OH)_2_ and CoO(OH), respectively. This process results in the increasement of anodic peak current by prompting the oxidation of Ni(OH)_2_ to NiO(OH) and CoO(OH) to CoO_2_. It is worth noting that the anodic peak currents display a linear dependence on glucose concentration (*C*_glucose_) ranging from 0–5 mM as shown in the inset of Fig. 3b. The linear fitting equation of *I*_pa_-*C*_glucose_ can be expressed as follows:

1 $$ {I}_{\mathrm{pa}}\left(\upmu \mathrm{A}\right)=11104\ \upmu \mathrm{A}+1353.8\ \upmu \mathrm{A}\cdotp {\mathrm{mM}}^{\hbox{-} 1}\ {C}_{\mathrm{glucose}}\left({\mathrm{R}}^2=0.9964\right) $$

The working area of the amorphous phase in our case was 1.0 cm^−2^, and the sensitivity of the amorphous samples was 1353.8 μA mM^−1^ cm^−2^. Furthermore, with the increasing glucose concentration, the cathodic peak currents *I*_pc_ were decreased gradually, which can be attributed to the consumption of Ni^3+^ and Co^4+^ in the electrooxidation of glucose. The detail catalytic oxidation reactions can be described as follows [41]:

2 $$ \mathrm{Ni}{\left(\mathrm{OH}\right)}_2+{\mathrm{OH}}^{\hbox{-}}\to \mathrm{Ni}\mathrm{O}\left(\mathrm{OH}\right)+{\mathrm{H}}_2\mathrm{O}+{\mathrm{e}}^{\hbox{-} } $$

3 $$ \mathrm{Ni}\mathrm{O}\left(\mathrm{OH}\right)+\mathrm{glucose}\to \mathrm{Ni}{\left(\mathrm{OH}\right)}_2+\mathrm{gluconolactone} $$

4 $$ \mathrm{Co}{\left(\mathrm{OH}\right)}_2+{\mathrm{OH}}^{\hbox{-}}\to \mathrm{Co}\mathrm{O}\left(\mathrm{OH}\right)+{\mathrm{H}}_2\mathrm{O}+{\mathrm{e}}^{\hbox{-} } $$

5 $$ \mathrm{CoO}\left(\mathrm{OH}\right)+{\mathrm{OH}}^{\hbox{-}}\to {\mathrm{CoO}}_2+{\mathrm{H}}_2\mathrm{O}+{\mathrm{e}}^{\hbox{-} } $$

6 $$ {\mathrm{CoO}}_2+\mathrm{glucose}\to \mathrm{CoO}\left(\mathrm{OH}\right)+\mathrm{gluconolactone} $$

In order to understand the electrochemical kinetic process during the electrooxidation of glucose on the Co-Ni hydroxide electrode, the CV curves of glucose oxidation as a function of scan rate were carried out in 0.5 M *C*_NaOH_ solution containing 5 mM glucose as shown in Fig. 3c. The redox peak current values (*I*_pa_ and *I*_pc_) increased with the increase of scan rate from 10 to 60 mV s^−1^, whereas the peak potentials were shifted negatively for *E*_pc_ and positively for *E*_pa_. These phenomena could be attributed to the increase of overpotential and kinetic limitation for amorphous Co-Ni hydroxide electrode toward glucose electrooxidation. As shown in Fig. 3d, the plot of *I*_pa_-*ν*^1/2^ in 0.5 M NaOH solution containing 5 mM glucose performed excellent linearity with the high correlation coefficient of 0.998, suggesting that the glucose oxidation which occurred on the Co-Ni hydroxide electrode is a diffusion controlled process [29].

### Amperometric Detection of Glucose

To evaluate the accurate electrocatalytic response of the glucose oxidation at the amorphous Co-Ni hydroxide electrode surface, amperometry technique was carried out in 20 mL stirred 0.5 M *C*_NaOH_ solution with the successive step addition of a known concentration of glucose at an applied potential of 0.36 V *vs* SCE (Fig. 4a). It can be easily found that a notable enhancement of current response was rapidly acquired after the glucose solution addition and reached a steady state within 5 s, suggesting a fast rate of oxidation reaction between glucose and redox sites of Co-Ni hydroxide electrode. The above phenomena reveal that Co-Ni hydroxide electrode performed a sensitive and fast response to the *C*_glucose_ variation under the alkaline conditions. As shown in Fig. 3b, the calibration curve of the response current as a function of glucose concentration demonstrates that the response currents increased linearly with the successive additions of glucose. The corresponding fitting plot reveals that the response curve can be divided into two distinctive linear ranges. The first linear range at low concentrations was observed from 0.00025 to 1 mM with a high correlation coefficient of 0.9994, and the linear fitting equation can be expressed as follows:

7 $$ {\mathrm{I}}_{\mathrm{pa}}\left(\upmu \mathrm{A}\right)=40.026\ \upmu \mathrm{A}+1911.5\ \upmu \mathrm{A}\cdot {\mathrm{mM}}^{-1}\ {C}_{glucose} $$

The second linear range at higher concentrations of glucose was from 1 to 5 mM with a linear correlation coefficient of 0.997, and the linear fitting equation can be expressed as follows:

8 $$ \mathrm{Ipa}\ \left(\upmu \mathrm{A}\right)=780.2\ \upmu \mathrm{A}+1397.5\ \upmu \mathrm{A}\cdot {\mathrm{mM}}^{\hbox{-} 1}{C}_{glucose} $$

From the fitting plot, the sensitivity of the sensor was calculated to be 1911.5 μA·mM^−1^ cm^−2^ at low concentrations of glucose and 1397.5 μA·mM^−1^ cm^−2^ at high concentrations of glucose. Thus, the result is similar to that calculated from the CV curves, confirming the high sensitivity of the amorphous Co–Ni(OH)_2_ nanostructures. The limit of detection (LOD) can be calculated by using the equation as follows [18, 29]:

9 $$ \mathrm{LOD}=3\upsigma /\mathrm{S} $$

where *σ* is the standard deviation of the background current obtained before the addition of the glucose and *S* is the slope of calibration plot of *I*-*C*_glucose_. The detection limit was estimated to be 0.12 μM in 0.5 M NaOH solution from a linear range at low glucose concentrations. Furthermore, it can be found that the response current Δ*I* (defined as the increment of current corresponding to the *C*_glucose_ increment of 1 mM) nearly kept a constant value with a *C*_glucose_ increase under the low *C*_glucose_ conditions, and the sensor exhibited a faster current response to the addition of glucose with higher sensitivity and linear correlation coefficient. But, when increasing the *C*_glucose_ to a high level, Δ*I* decreased. If the *C*_glucose_ exceeds a certain level, Δ*I* decreased dramatically resulting in the linear relationship being destroyed. Under lower *C*_glucose_ conditions, the amount of surface active sites is more than that of glucose molecules, which make the response current to be enhanced linearly with the successive additions of glucose. With the *C*_glucose_ increasing, more and more active sites are covered by the glucose molecules, resulting in some glucose molecules inaccessible for oxidation and making Δ*I* decrease notably.

**Fig. 5:**
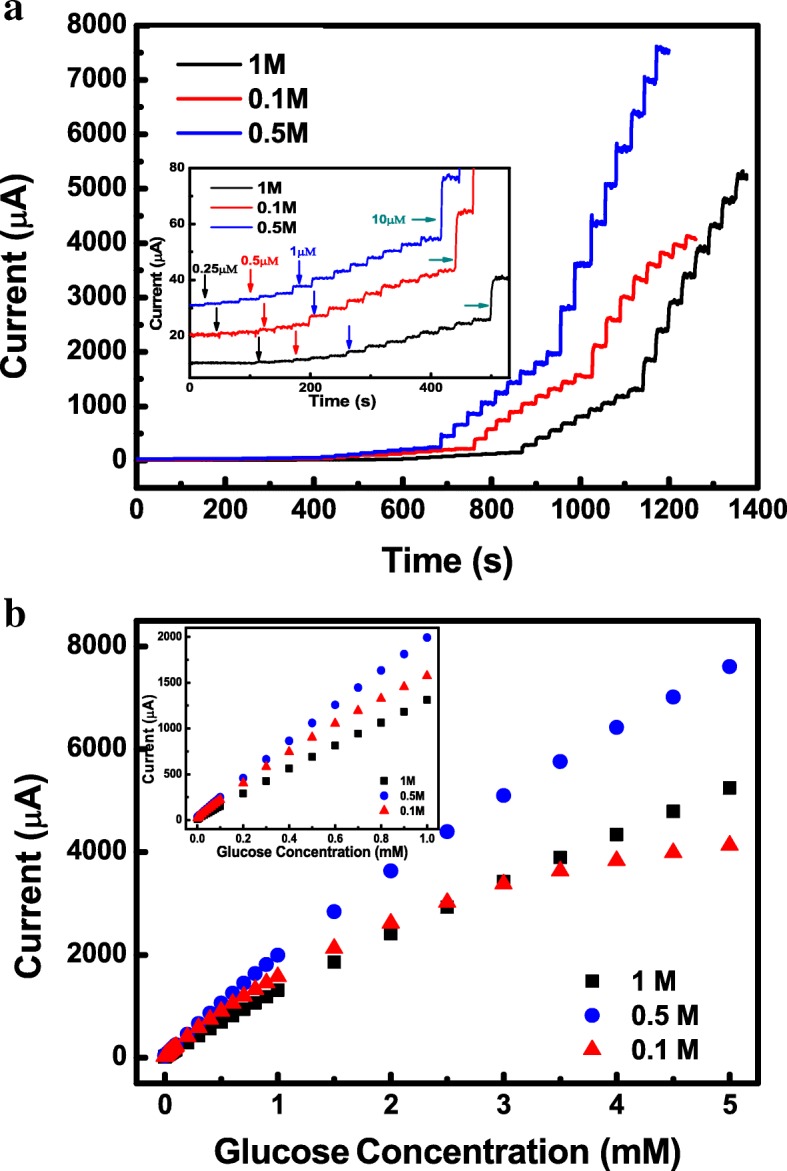
Current response to glucose in different concentrations of NaOH. The current-time response (**a**) and current-*C*_glucose_ curves (**b**) for Co-Ni hydroxide electrode acquired in 0.1 M, 0.5 M, and 1 M NaOH.

As the electrochemical activity is highly dependent on the OH^−^ anion concentrations (C_OH_^−^) under the alkali conditions, the glucose detection performance of Co-Ni hydroxide electrode is possibly influenced by the C_OH_^−^ of alkali electrolyte [42]. In order to get optimal concentration, the effect of C_OH_^−^ on amperometric response toward glucose oxidation for Co-Ni hydroxide was investigated in NaOH solution with several concentrations at an applied potential around the anodic peak (Fig. 5). A known concentration of glucose ranging from 0.00025 to 5 mM was consecutive stepwise added into 20 mL stirred NaOH solution with three different concentrations of 0.1, 0.5, and 1.0 M. All the amperometry curves in different *C*_NaOH_ exhibited rapid current response to the addition of glucose as shown in Fig. 5a. The plots of the response current as a function of glucose concentration in 0.1 M, 0.5 M, and 1 M NaOH solution are shown in Fig. 5b. It can be observed that the amorphous Co-Ni hydroxide for the glucose detection in 0.5 M NaOH solution displayed an optimal glucose-sensing performance with a higher sensitivity, a lower detection limit, and a wider linear range with high correlation coefficient compared to that of in 0.1 and 1 M NaOH. Considering that high sensitivity, low LOD, wide linear concentration range, and high correlation coefficient are the benefit for enhanced accuracy and decreased deviation of glucose detection as the sensor materials applied in practical applications, the OH^−^ anion concentration of 0.5 M was chosen as the optimal working electrolyte for glucose detection in our studies.

**Fig. 6:**
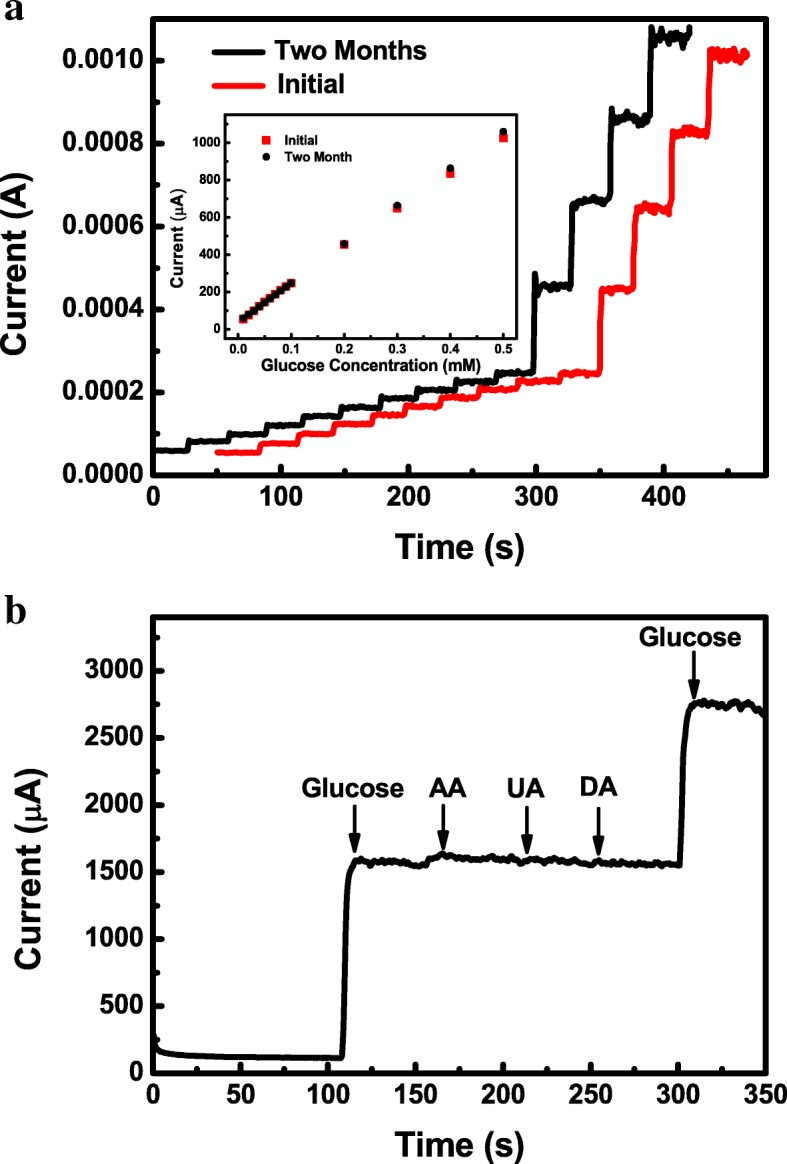
The stability and selectivity of amorphous electrode for electrochemical sensors. **a** The current response to glucose with the successive step addition in 20 mL stirred electrolyte of 0.5 M NaOH over 2 months. **b** The amperometric current-time (i-t) curve of the glucose oxidation with other carbohydrates on the Co-Ni hydroxide electrode acquired in 20 mL stirred electrolyte of 0.5 M NaOH with the successive step addition of 1 mM glucose and 0.1 mM common interferents.

The long-term stability, another critical factor for electrochemical sensors in practical applications, was investigated by examining the amperometric response to glucose with the successive step addition in 20 mL stirred 0.5 M NaOH electrolyte. As shown in Fig. 6a, after 2 months, no decrement of the current response toward the glucose electrooxidation compared with its initial response current can be observed. The sensitivity retained 103% of the initial value even after 2 months, implying excellent long-term stability of glucose sensor based on amorphous Ni-Co hydroxide. Furthermore, Table 1 shows a comparative sensing performance evaluation of our fabricated glucose sensor with the other nickel-based and cobalt-based non-enzymatic glucose sensors reported by previous literature on the sensitivity, linear range, detection limit, and long-term stability. The glucose detection performances of fabricated amorphous Co-Ni hydroxide sensor obtained in our study are comparable and even superior to most nickel-based and cobalt-based non-enzymatic glucose sensors reported elsewhere, especially, our sensor shows remarkable long-term stability, guaranteeing its optimal potential in real biological sample analysis.

**Table 1:** Comparison of the sensing performance of non-enzymatic amorphous Co-Ni hydroxide glucose sensor with other reported nickel-based and cobalt-based non-enzymatic glucose sensors

Electrode	Sensitivity (μA mM^−1^ cm^−2^)	Linear range (mM)	Correlation coefficient	Detection limit (μM)	Stability	Refs.
Amorphous Ni(OH)_2_ nanoboxes/GCE^a^	487.3	0.0005~5	0.998	0.07	30 days, 95%	30
CNFS/Co(OH)_2_	6800	0.01~0.12	0.999	5	30 days, 98.5%	38
Co_3_O_4_ nanoclusters	1377	0.088~7	0.99	26	30 days, 96.1%	[43]
CoOOH nanosheet arrays/Ti foil	562.8	0.003~1.109	0.995	1.37	—	22
Ni(OH)_2_ flakes/Ni foam	2617	0.0025~1.050	0.9989	2.5	30 days, 93.8%	18
NiO nanosheets/GCE	1138	0.001~0.4	0.999	0.18	10 days, 94%	17
NiOOH nanosheets	2697	0.0005–0.117		0.2	0.9975	
Ni-Co hydroxide	122.45	0.025~3.7	0.989	–	–	27
Ni-Co oxide nanowrinkles/rGO^b^	548.9	0.005-8.56	0.999	2	30 days, 91.5%	41
Amorphous Co-Ni hydroxide	1911.5	0.00025~5.0	0.9994	0.12	60 days, 102%	This work

^a^Glassy carbon electrode

^b^Reduced graphene oxide

Generally, the electrochemical sensing performances of a sensor for glucose detection depend significantly on the intrinsic sensing activity of sensor material and the number of active sites. For hydroxide-based electrochemical glucose sensors, the intrinsic sensing activity is usually tightly determined by the material composition, crystal structure, defects, redox couples, electronic conductivity, and charge transfer capability; the number of active sites is largely related to the material morphology, particle size, and surface microstructure. Based on the above consideration, the outstanding glucose sensing ability of the amorphous Co–Ni(OH)_2_-based sensor primarily comes from the following factors. The morphology of pure and ravine-like surfaces and three-dimensional (3D) nanostructure is the first factor for their promising electrochemical sensing activity. The pure surfaces, resulting from a chemically clean reaction environment preparation, are the benefit for the enhanced efficiency of amorphous Co–Ni(OH)_2_ nanostructures interacting with glucose molecular. The ravine-like surfaces and 3D nanostructures offer high specific surface area, resulting in the number of active sites increased, which can significantly improve the sensing activity. The second fact is the homo-incorporation of a second metal element for metal hydroxide, providing an easier accessible pathway for the intercalation and deintercalation of charges, which promotes the conversion of Ni^2+^/Ni^3+^ and Co^2+^/Co^3+^/Co^4+^. The fast Ni^2+^/Ni^3+^ and Co^2+^/Co^3+^/Co^4+^ conversion rate means that the amount of NiO(OH) and CoO_2_ active sites can be kept in a sufficient value to make glucose oxidized fast and adequately even in the presence of high *C*_glucose_. Lastly, the self-assembled amorphous phase plays a crucial role in improving the electronic conductivity, charge transfer capability, and longevity of the sensor. The amorphous phase is characteristic of long-range disorder, short-range ordered structure, lots of defects, and unsaturated ligand atoms, which lead to a significant improvement in the electronic conductivity of the amorphous materials and an increment in the number of electrochemically active sites. Meanwhile, the self-assembly maintains the structural continuity which significantly enhances the electronic conductivity and electrical contact between the nanostructures and the substrate. Moreover, during redox reaction, electrostatic interactions between metal ions are thus uniformly distributed or isotropic in the amorphous structure because of the long-range disordered nature of the amorphous phase. The electrostatic force, caused by changing the charge during the conversion of Ni^2+^/Ni^3+^ and Co^2+^/Co^3+^/Co^4+^, can fully relax and be released in the amorphous structure resulting in the structure of the amorphous material keeping stable. In other words, the amorphous phase sensors will perform considering the long-term stability during glucose detection.

### Interference Studies

The above results indicate that the amorphous Co-Ni hydroxide displays an excellent glucose-sensing behavior with high sensitivity, wide linear range, and long-term stability in the absence of other interfering species under the alkali conditions. However, it is known that some easily oxidative interfering species, such as ascorbic acid (AA), uric acid (UA), and dopamine (DA), are usually coexisting with glucose in the human serum. The selectivity of glucose detection, related to its response for glucose in the presence of other competing species, is another important factor and challenge for electrochemical sensors in practical applications. The influences of various interfering species, such as AA, UA, and DA, on glucose sensing of the amorphous Co-Ni hydroxide electrode were studied by using amperometry technique. The physiological level of glucose in human blood is about 3–8 mM, which is substantially higher than the concentrations of interfering species such as AA (0.1 mM), UA (0.1 mM), and DA (0.1 mM). Hence, the interference test of the modified electrode toward glucose oxidation was carried out in 20 mL stirred electrolyte of 0.5 M NaOH by the successive step additions of 1 mM glucose and 0.1 mM AA, UA, and DA. The corresponding amperometric responses are exhibited in Fig. 6b. A small rise of the response current can be observed with the addition of AA which induces a small rise of the current, but the increment is much smaller than that for glucose (about 2.5%). Meanwhile, there are no obvious current responses observed in the addition of DA and UA. As a result, the amorphous Co-Ni hydroxide electrode displayed negligible current responses toward the interfering species in comparison with that of glucose, suggesting high selectivity toward glucose for the prepared amorphous Co-Ni hydroxide as non-enzymatic electrochemical sensor and excellent applicability to the real sample analysis.

### Real Sample Analysis

In order to verify the commercial reliability and applicability of the modified glucose sensor, the glucose concentration in the real sample was detected using the amperometric method. Amperometric response of the amorphous Co-Ni hydroxide electrode toward glucose oxidation was monitored with the successive step addition of 0.1 mM glucose to 20 mL stirred 0.5 M NaOH solution containing serum samples. As shown in Table 2, the non-enzymatic glucose sensor displays recoveries in the range of 97.92–100.33% and 2.66–3.99% relative standard deviation (RSD), implying that the as-synthesized amorphous Co-Ni hydroxide glucose sensor holds great potential in real biological sample analysis.

**Table 2:** Real sample analysis of glucose in human serum samples

Glucose added (mM)	Glucose found^a^ (mM)	Recovery (%)	RSD^b^ (%)
0.1	0.10068	100.68	2.51
0.2	0.20346	101.73	1.78
0.3	0.29967	99.89	1.43

^a^Standard deviation method

^b^Relative standard deviation with three replicative measurements

## Conclusions

A facile approach has been demonstrated for the synthesis of amorphous Co-Ni hydroxide with a homogeneous architecture by a simple and chemically clean electrochemical deposition route. The electrocatalytic activities of fabricated amorphous samples toward non-enzymatic glucose sensors have been investigated under the alkaline conditions. The as-synthesized amorphous Co-Ni hydroxide sensor exhibits a superior biosensing performance toward glucose oxidation with high sensitivity of 1911.5 μA·mM^−1^ cm^−2^ and low-level detection limit of 0.12 μM at the lower concentration of glucose, wide linear range from 0.25 to 5 mM, fast response within 5 s, and super long-term stability and excellent selectivity in 0.5 M NaOH solution. These results reveal the great potential of amorphous Co-Ni hydroxide as glucose sensor materials for use in non-enzymatic glucose detection.

## Abbreviations

3D: Three-dimensional
AA: Ascorbic acid
CV: Cyclic voltammetry
DA: Dopamine
*E*_pa_: The potentials of anodic peak
*E*_pc_: The potentials of cathodic peak
HRTEM: High-resolution transmission electron microscope
*I*_pa_: Rhe anode peak current
RSD: Relative standard deviation
SAED: Selected area electron diffraction
SCE: Saturated calomel electrode
SEM: Scanning electron microscope
TEM: Transmission electron microscope
UA: Uric acid
XPS: X-ray photoelectron spectroscopy
